# Medical Society of the County of Erie

**Published:** 1898-07

**Authors:** F. C. Gram


### Society Proceedings.

### MEDICAL SOCIETY OF THE COUNTY OF ERIE.

*Semi-annual meeting, held June 14, 1898.*

Reported by F. C. GRAM, M. D., Secretary.

PRESIDENT LUCIEN HOWE called the semi-annual meeting of the Medical Society of the County of Erie to order at 10 o'clock a. m., Tuesday, June 14, 1898, in the parlors of the Buffalo Academy of Medicine.

The secretary read the minutes of the annual meeting and of the special meeting, held May 11, 1898, which were then approved as read.

Dr. W. W. POTTER, chairman of the committee on membership, reported favorably upon the following applicants for membership: Drs. L. E. McC. Pomeroy, Francis E. Fronczak, Carro Julia Cummings, John J. Finnerty, F. W. McGuire and John H. Daniels. They were thereupon elected members. The applications of Drs. N. L. Burnham, James W. Charters and Christ W. Weinbach are pending and will be acted upon when the requirements have been fulfilled.

Applications for membership were received from Drs. John H. Grant, J. C. Clemesha, H. R. Gaylord, A. L. Weil, Emil Lustig and Mary Clayton.

Referred to committee on membership.

Dr. COAKLEY, chairman of the board of censors, submitted the following report:

*To the Erie County Medical Society, Buffalo, N. Y.:*

GENTLEMEN:—The board of censors respectfully presents to you its semi-annual report for the six months ending June 14, 1898, as follows:

In accordance with the resolution adopted at the last session of the Society this board has employed as its counsel for the prosecution of illegal practitioners in this county, Messrs. Tracy C. Becker and Marc W. Comstock. The Society is indebted to these gentlemen for efficient and successful efforts in the prosecution of all matters entrusted to them.

Several cases of alleged nonregistration and illegal practice have been presented to this board during the past six months and all such instances thoroughly investigated. One case was sufficiently definite and well proven to authorise a prosecution, to-wit: the case against Joseph Rutkowski, a versatile gentleman, who seemed to be acting as barber, dentist and physician. He advertised in a number of papers. He was arraigned before Judge King and the case tried by our counsel, with the result that Rutkowski was held for the grand jury. The grand jury found an indictment and on May 3d Rutkowski pleaded guilty to the charge against him. Judge Titus suspended sentence upon the promise of the offender to discontinue his illegal practice.

We beg to report further that our counsel have made a complete list of all registered physicians in the county of Erie. This list will be compared with the list of physicians in the new city directory, soon to be issued, and all nonregistered physicians investigated. The active efforts of this board in the past, and the publicity given to this work in January last, apparently have had a very salutary effect, the number of cases for investigation having been much less the last six months than previously.

We beg, in closing our report, to express our appreciation of the hearty coöperation rendered us and our counsel by the district attorney's office.

Respectfully submitted,

J. B. COAKLEY, *Chairman*,
IRVING W. POTTER,
THOMAS F. DWYER,
CHARLES E. CONGDON.

The report was received and placed on file.

The treasurer, Dr. EDWARD CLARK, submitted a list of names of members in arrears with their dues, in accordance with a resolution of this society at the last annual meeting. He also explained the efforts he had made toward collecting them.

The chairman ruled that it was unnecessary to lay the details of the report before the society.

Dr. W. W. POTTER moved to hear the report of the treasurer relative to the resolution adopted at the annual meeting. Seconded by Dr. J. C. Brown and adopted.

Treasurer CLARK then completed his report, which, on motion of Dr. Coakley was discussed, and Dr. Coakley then moved that the treasurer continue his efforts to collect the arrears, giving all another opportunity to pay. Adopted.

Treasurer CLARK wanted to know what to do with those members who absolutely refuse to pay, in violation of the by-laws of this society, as well as with those who are advertising in the daily papers.

The president ruled that the motion adopted at the annual meeting and the by-laws of this society made the necessary provisions.

Dr. GEORGE W. ABBOTT stated that he had been a member of this society for forty-five years, paying his dues for the greater part of that period. However, for the past few years he had been unable to meet them and craved the indulgence of the society.

On motion of Dr. W. W. POTTER the accumulated dues of Dr. Abbott were remitted.

Dr. LEON F. HARVEY, of Denver, Colo., presented his resignation as a member, which was accepted.

Dr. J. G. THOMPSON moved the following resolutions, which were adopted:

WHEREAS, The Medical Society of the County of Erie desires to express its appreciation of the sacrifices made by members of the society who have voluntarily given up the comforts of home to endure the privations and perils of active service in the army. Therefore,

*Resolved*, That we hereby send to Drs. Albert H. Briggs, Eugene A. Smith, Clark F. Bruso, E. L. Ruffner, Harry Mead and John D. Howland a hearty greeting with our admiration of their patriotism in answering so unselfishly and so promptly their country's call.

*Resolved*, That it shall be our endeavor to have their absence result in as small a professional loss to them as possible, and that we consider it becoming when patients of theirs come to us who remain here to receive such patients only in professional trust as we would from some brother physician whose practice has been for a time left in our charge.

*Resolved*, That we assure to these absent members, as far as we can, the reëstablishment, on their return, of all their former professional relations.

On motion of Dr. COAKLEY the sum of $20 was ordered paid, as the expense incurred by the censors for detective work relative to securing evidence against illegal practitioners.

Dr. ROSWELL PARK then addressed the society on The modern treatment of gun-shot wounds.

The subject was further discussed by Drs. D. W. Harrington, W. W. Potter, J. B. Coakley and J. G. Thompson, all of whom spoke from personal army experience. A vote of thanks was tendered Dr. Park and the society adjourned at 12.45 o'clock.

